# Low Geriatric Nutritional Risk Index Is Associated with Poorer Prognosis in Elderly Diffuse Large B-Cell Lymphoma Patients Unfit for Intensive Anthracycline-Containing Therapy: A Real-World Study

**DOI:** 10.3390/nu13093243

**Published:** 2021-09-17

**Authors:** Tzer-Ming Chuang, Yi-Chang Liu, Hui-Hua Hsiao, Hui-Ching Wang, Jeng-Shiun Du, Tsung-Jang Yeh, Yuh-Ching Gau, Ya-Lun Ke, Ching-I Yang, Ching-Ping Lee, Chin-Mu Hsu, Shih-Feng Cho

**Affiliations:** 1 Division of Hematology & Oncology, Department of Internal Medicine, Kaohsiung Medical University Hospital, Kaohsiung Medical University, Kaohsiung 807, Taiwan; benjer6@gmail.com (T.-M.C.); ycliu@cc.kmu.edu.tw (Y.-C.L.); huhuhs@kmu.edu.tw (H.-H.H.); joellewang66@gmail.com (H.-C.W.); ashiun@gmail.com (J.-S.D.); aw7719@gmail.com (T.-J.Y.); cheesecaketwin@gmail.com (Y.-C.G.); a9601082@gmail.com (Y.-L.K.); febeey0118@gmail.com (C.-I.Y.); ping890218@gmail.com (C.-P.L.); e12013@gmail.com (C.-M.H.); 2 Faculty of Medicine, College of Medicine, Kaohsiung Medical University, Kaohsiung 807, Taiwan; 3 Center for Cancer Research, Kaohsiung Medical University, Kaohsiung 807, Taiwan; 4 Specialist Nurse and Surgical Nurse Practitioner Office, Kaohsiung Medical University Hospital, Kaohsiung Medical University, Kaohsiung 807, Taiwan

**Keywords:** diffuse large B cell lymphoma, elderly patients, Geriatric Nutritional Risk Index

## Abstract

Nutritional assessments, including the Geriatric Nutritional Risk Index (GNRI), have emerged as prediction tools for long-term survival in various cancers. This study aimed to investigate the therapeutic strategy and explore the prognostic factors in the elderly patients (≥65 years) with diffuse large B cell lymphoma (DLBCL). The cutoff value of the GNRI score (92.5) was obtained using the receiver operating characteristic curve. Among these patients (*n* = 205), 129 (62.9%) did not receive standard R–CHOP chemotherapy. Old age (≥80 years), poor performance status, low serum albumin level, and comorbidities were the major factors associated with less intensive anti-lymphoma treatment. Further analysis demonstrated that a lower GNRI score (<92.5) was linked to more unfavorable clinical features. In the patients who received non-anthracycline-containing regimens (non-R–CHOP), multivariate analysis showed that a low GNRI can serve as an independent predictive factor for worse progression-free (HR, 2.85; 95% CI, 1.05–7.72; *p* = 0.039) and overall survival (HR, 2.98; 95% CI, 1.02–8.90; *p* = 0.045). In summary, nutritional evaluation plays a role in DLBCL treatment and the GNRI score can serve as a feasible predictive tool for clinical outcomes in frail elderly DLBCL patients treated with non-anthracycline-containing regimens.

## 1. Introduction

Diffuse large B cell lymphoma (DLBCL) is the most common type of non-Hodgkin lymphoma (NHL), accounting for approximately 35% of newly diagnosed lymphoma cases, with the average age of diagnosis exceeding 65 years [1]. Due to the aggressive nature of the disease and physical frailty of elderly patients, poorer progression-free survival (PFS) and overall survival (OS) have been observed in several studies [2,3]. Since the introduction of rituximab in combination with conventional chemotherapy, significant improvement of treatment outcomes has been observed [4,5]. Nevertheless, treatment strategies are often highly individualized in elderly patients, who are more likely to suffer from life-threatening chemotherapy-related toxicity due to chronic or weakness-associated diseases [6]. As a result, nationwide database analyses in many countries showed that more than 50% of the elderly patients did not receive anthracycline-containing regimens as the first-line treatment [7,8,9].

The international prognostic index (IPI) and the age-adjusted IPI (aaIPI) have been widely used as prognostic tools in DLBCL [10]. These models were developed based on patients receiving anthracycline-containing regimens, and more than half of the participants were under 60 years old, limiting their application in elderly patients receiving a less intensive therapy. In the recent years, nutritional status and comorbidity assessment tools have emerged as critical factors of predicting the long-term prognosis in these subgroups [11,12,13,14,15,16,17]. For example, previous studies showed that the Charlson Comorbidity Index (CCI), a standardized scoring system to predict mortality with respect to the weight of comorbidities, can predict the clinical outcome in lymphoma patients [14,15]. Parameters of the nutritional status, including the serum albumin level, body weight, and body mass index (BMI), were also explored as predictors [11,12,14,16,17,18,19,20,21,22]. Previous studies revealed that skeletal muscle and fat mass wasting identified by computed tomography (CT) are valuable for predicting the clinical outcomes in DLBCL patients [23,24]. The evidence demonstrates that high BMI is misleading because it does not account for body composition, and many patients with cancer may present with a normal or high BMI but have severe muscle depletion [19,20]. On the other hand, the serum albumin level is also reported to be a prognostic factor in DLBCL patients [12,21,22]. However, the serum albumin level is now recognized as being significantly influenced by inflammation; it is also a poor measure of the nutritional status and more likely suggestive of disease severity, not the nutritional status [25,26].

The Geriatric Nutritional Risk Index (GNRI) is a novel nutritional scale consisting of two major objective nutritional parameters, serum albumin level and body weight, to predict the mortality risk in elderly patients with different cancer types [27,28]. Several studies have demonstrated the GNRI to be a better prognostic factor than the serum albumin level, body weight, or BMI alone in hematologic malignancies [29,30,31]. Regarding lymphoma, the GNRI was associated with long-term survival in DLBCL patients [13,16,17,18]. However, the impact of the GNRI on different treatment strategies has not been elucidated.

Our prior studies have revealed that advanced age, high aaIPI score, and bone marrow (BM) involvement are associated with poorer survival and that the individual performance status and CCI affect the initial treatment strategies [7,32]. However, their correlation with the nutritional status was not evaluated. Hence, we investigated whether the nutritional status influences treatment strategies and prognosis. This study aimed to evaluate the impact of comorbidities on various therapeutic interventions and explore the prognostic value of the GNRI in elderly DLBCL patients in a real-world setting.

## 2. Materials and Methods

### 2.1. Patient Population

This is a retrospective study conducted at the Kaohsiung Medical University Hospital in Taiwan. The medical records of elderly patients (≥65 years) with newly diagnosed and pathologically proven DLBCL between 2010 and 2019 were reviewed and collected. Patients with primary central nervous system and transformed DLBCL, as well as with human immunodeficiency virus (HIV) infection, were excluded. Anthropometric data (height, weight, and BMI), age, sex, medical history, Eastern Cooperative Oncology Group Performance Status (ECOG PS), extranodal involvement, Ann Arbor stage, aaIPI, B symptoms, complete blood cell count, serum albumin (Alb), serum creatinine (Cr), lactate dehydrogenase (LDH), and β2-microglubulin (B2M) were collected. BM involvement was determined in the pathological review of the BM biopsy or visual interpretation of marrow fluorine-18 fluorodeoxyglucose (FDG) uptake during whole-body staging positron emission tomography/computed tomography (PET/CT) scanning by nuclear medicine radiologists. The CCI, which is calculated on the basis of 19 items except for “lymphoma,” was also used to evaluate the impact of comorbidities on clinical outcomes [33,34,35].

The protocol for this study, including data collection, was conducted in accordance with the principles of the Declaration of Helsinki and approved by the Institutional Review Board of Kaohsiung Medical University Hospital (KMUH-IRB-E(I)-20200014).

### 2.2. Treatment of DLBCL

To treat DLBCL, the standard regimen is the combination of rituximab and conventional chemotherapy. The anthracycline-containing (R–CHOP) regimen includes 375 mg/m^2^ rituximab on day 1, 750 mg/m^2^ cyclophosphamide on day 2, 50 mg/m^2^ doxorubicin on day 2, 1.4 mg/m^2^ vincristine (up to the maximal dose of 2 mg) on day 2, and 40 mg/m^2^ prednisolone for five days. The patients were treated for six–eight planned treatment courses. Doxorubicin was not prescribed to the patients receiving the R–COP treatment, but the remaining therapeutic agents and schedule were the same as in R–CHOP. Treatment choices, including steroid administration, rituximab infusion, and supportive care, were used if the patients could not tolerate intensive chemotherapy with the curative intent. The therapeutic intervention for each patient was determined by the physicians after discussion in a multidisciplinary team meeting and performed after the intervention was thoroughly explained to the patient and their family.

### 2.3. Geriatric Nutritional Risk Index

The GNRI was calculated based on body weight (BW) and serum Alb using the following equation: 14.89 × Alb (g/dL) + 41.7 × (BW/ideal BW). The ideal BW was defined as 22 × (body height (m))^2^. BW/ideal BW was defined as 1 when the patient’s BW exceeded the ideal BW [27].

### 2.4. Clinical Outcomes and Statistical Analysis

The response to treatment was evaluated based on the International Workshop criteria [36]. PFS was defined as the date of diagnosis to the date of lymphoma progression or death from any cause. OS was defined as the duration from the date of diagnosis to the date of death from any cause. All the patients in this study were followed by maintaining close contact either at home or at the hospital to identify the date and the cause of death.

The PFS and OS curves were plotted using the Kaplan–Meier method and compared using the logrank test. Chi-squared tests and Mann–Whitney U tests were applied to evaluate the differences in the categorical and quantitative data between the groups. Correlations between two variables were assessed using the Pearson correlation. A receiver operating characteristic (ROC) curve was used to estimate the discriminative cutoff value of the GNRI with the maximum Youden index (Youden index = sensitivity + specificity − 1). We used multivariable logistic regression to examine the association between patient clinical factors and binary choices of treatment. The hazard ratios (HRs) and 95% confidence intervals (CIs) were calculated using multivariate Cox regression to investigate the relative risks. Additionally, we performed Cox regression in univariate and multivariate analyses to examine the risk factors for early mortality. Variables with a *p*-value less than 0.05 in the univariate analyses were included in the multivariate analyses. All the statistical analyses were based on two-sided hypothesis tests with a significance level of the *p*-value  <  0.05 and performed with SPSS (SPSS Inc., 233 South Wacker Drive, IL, USA) version 25.

## 3. Results

### 3.1. Patients’ Characteristics and Treatment Strategies

There were 225 elderly patients with DLBCL diagnosed between 2010 and 2019. After excluding patients with primary central nervous system DLBCL (*n* = 15), transformed DLBCL (*n* = 2), and incomplete data (*n* = 3), the clinical data of 205 patients (including 107 men and 98 women) with the median age of 75 years (range, 65–96 years) at the time of diagnosis were collected for this study. The median PFS and OS of the total population were 9.3 and 13.5 months, respectively. The median OS of the patients between 65 and 69, 70 and 79, and ≥80 was 35.1, 15.9, and 4.3 months, respectively. The median GNRI was 94 (range, 40–115). The detailed demographic data of these patients are listed in Table 1.

Of the 205 patients, 182 patients received systemic chemotherapy, including the R–CHOP regimen (*n* = 76), and 23 (11.2%) patients received steroid monotherapy (Table S1). Most patients (*n* = 69) who underwent the R–CHOP treatment received dose reduction of anthracycline during treatment courses, with the mean cumulative dose of anthracycline of 155.1 mg/m^2^, the average dose of 27.5 mg/m^2^ per cycle, and the average number of cycles of 5.6 ± 2.1 (range, 1–8). Compared with the patients receiving the R–CHOP treatment, the non-R–CHOP group was significantly older, with a poorer performance status and lower serum Alb levels. Additionally, the non-R–CHOP group had significantly high percentages of at least one comorbidity or multiple comorbidities and a lower median GNRI score (Table 1).

The initial treatment strategy was also analyzed. In the very old age (≥80 years) patients or those with poorer performance scores (ECOG PS 2–4), a significantly higher percentage received a less intensive therapy, including the R–COP regimen or steroid monotherapy (Table 2). In the multivariable analysis, age ≥80 years (odds ratio (OR), 3.48; *p* = 0.004), poorer performance status (ECOG PS ≥ 2) (OR, 3.51; *p* = 0.006), low serum albumin level (OR, 2.59; *p* = 0.020), and at least one comorbidity (CCI ≥ 1) (OR, 3.08; *p* = 0.002) were all associated with a higher likelihood of receiving non-R–CHOP treatments (Table 3). 

### 3.2. Assessment of the Nutritional Status

According to the ROC analysis, the GNRI < 92.5 was defined as a nutritional risk (Figure S1). The number of patients categorized into the nutritional risk groups using the GNRI ≤ 92.5 was 87 (44.2%) (Table 1). The patients with a poorer nutritional status (GNRI ≤ 92.5) were significantly older (median age, 76 years vs. 75 years, *p* = 0.003), had a poorer performance status (ECOG PS ≥ 2, 50.6% vs. 21.4%, *p* < 0.001), had more advanced disease stages (Ann Arbor stage III/IV, 79.8% vs. 51.7%, *p* < 0.001), had a higher risk status (aaIPI ≥ 2, 72.5% vs. 39.1%, *p* < 0.001), and had higher serum LDH levels (median, 274 IU/dL vs. 199 IU/dL, *p* < 0.001). Fewer patients received R–CHOP as their first-line treatment in the low GNRI group (23.0% vs. 47.9%, *p* < 0.001). Between the high GNRI and low GNRI groups, no significant difference was found in the CCI ≥ 1 category (55.8% vs. 54.7%, *p* = 0.875) or a higher CCI score category (CCI ≥ 3, 15.1% vs. 15.4%, *p* = 0.958) (Table 4).

### 3.3. Outcome Analysis

**Figure 1:**
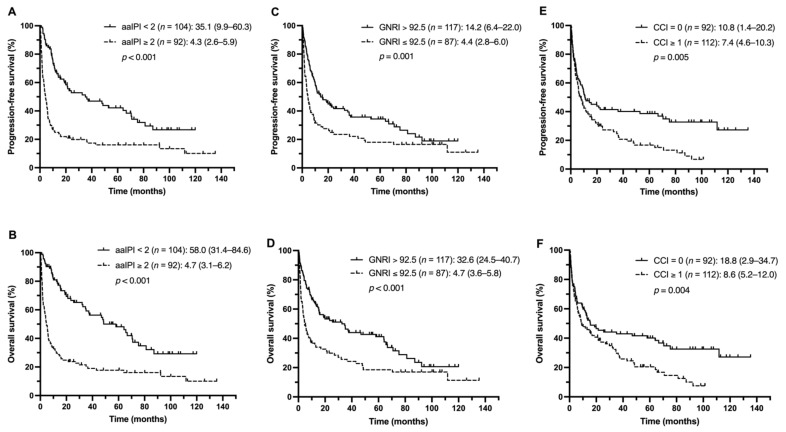
PFS and OS of the study population stratified by the aaIPI (**A**,**B**), GNRI (**C**,**D**), and CCI (**E**,**F**).

Overall, the patients with low aaIPI scores (aaIPI < 2) had significantly better PFS and OS (Figure 1A,B). When the patients were stratified by the nutritional status, the low GNRI group had the median PFS and OS of 4.4 and 4.7 months, respectively, which were significantly shorter than those of the high GNRI group (14.2 months and 32.6 months, with *p*-values of 0.001 and < 0.001, respectively) (Figure 1C,D). In addition, the patients with the CCI ≥ 1 had shorter median PFS and OS than the patients with the CCI = 0 (PFS, 7.4 vs. 10.8 months, *p* = 0.005; OS, 8.6 vs. 18.8 months, *p* = 0.004) (Figure 1E,F).

**Figure 2:**
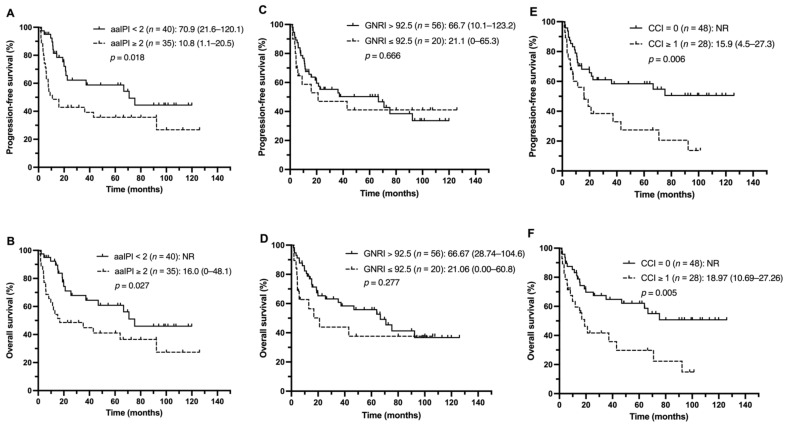
PFS and OS of the R–CHOP group stratified by the aaIPI (**A**,**B**), GNRI (**C**,**D**), and CCI (**E**,**F**).

**Figure 3:**
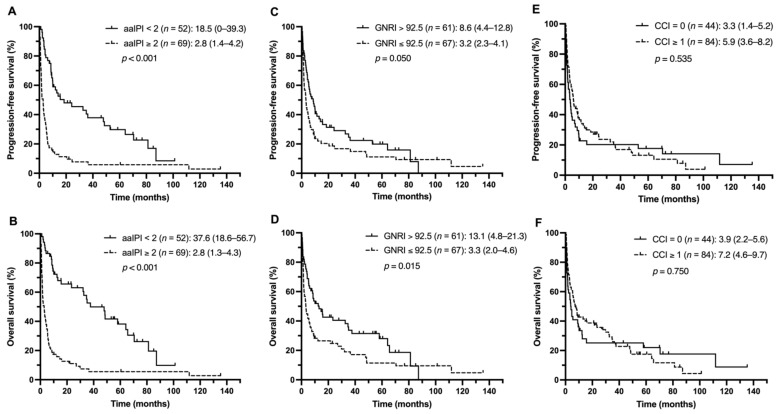
PFS and OS of the non-R–CHOP group stratified by the aaIPI (**A**,**B**), GNRI (**C**,**D**), and CCI (**E**,**F**).

Next, the impact of aaIPI scores, nutritional status, and comorbidities on clinical outcomes among the different treatment groups was investigated. The patients with lower aaIPI scores (<2) had more favorable PFS and OS in both the R–CHOP and non-R–CHOP groups (Figure 2A,B and Figure 3A,B). In the R–CHOP group, no significant difference was observed when comparing low and high GNRI scores (PFS, 21.1 vs. 66.7 months, *p* = 0.666; OS, 21.1 vs. 66.7 months, *p* = 0.277) (Figure 2C,D). Worse median PFS and OS were found in the CCI ≥ 1 group than in the CCI = 0 group (PFS, 15.9 months vs. not reached, *p* = 0.006; OS, 18.9 months vs. not reached, *p* = 0.005) (Figure 2E,F). In the non-R–CHOP treatment group, the median PFS and OS were significantly shorter in the low GNRI group than in the high GNRI group (PFS, 3.2 vs. 8.2 months, *p* = 0.050; OS, 3.3 vs. 13.1 months, *p* = 0.015) (Figure 3C,D). No statistically significant difference was observed between the CCI ≥ 1 and CCI = 0 groups (PFS, 5.9 vs. 3.3 months, *p* = 0.535; OS, 7.2 vs. 3.9 months, *p* = 0.750) (Figure 3E,F).

A total of 143 patients died during follow-up, including 84 patients who died from lymphoma progression and 24 patients who died from treatment-related toxicity. No significant difference in the cause of death was found between the patients with or without anthracycline regimens (*p* = 0.989) (Table S2). Importantly, 61 patients (29.8%) died within 120 days of diagnosis. Among these patients, 9, 21, and 31 were aged 65–69, 70–79, and ≥80 years, respectively. The majority of the patients (*n* = 39, 63.9%) died due to rapid progression or complications of lymphoma, and nine (14.8%) died from treatment-related toxicity. Multivariate Cox regression analysis revealed that advanced age (≥80), a high-risk status (aaIPI ≥ 2), and bone marrow involvement were independent risk factors for early mortality (Table S3).

### 3.4. Identification of Prognostic Factors for Survival

The prognostic factors for PFS and OS were evaluated. The results of the multivariate analysis revealed that a poorer performance status (ECOG PS ≥ 2) and high-risk status (aaIPI ≥ 2) were associated with adverse PFS in the R–CHOP group. CCI ≥ 1 only showed a significant result in the univariate analyses (*p* < 0.05), and low GNRI did not show any significance. In the non-R–CHOP group, a high-risk status (aaIPI ≥ 2), BM involvement, abnormal serum B2M, and low GNRI (GNRI ≤ 92.5) were independent risk factors for adverse PFS (Table 5).

Regarding the analysis of OS, a poorer performance status (ECOG PS ≥ 2) and high-risk status (aaIPI ≥ 2) were linked to adverse OS in the R–CHOP group in the multivariate analysis. In the non-R–CHOP group, a poorer performance status (ECOG PS ≥ 2), BM involvement at diagnosis, abnormal serum B2M level, and low GNRI (GNRI ≤ 92.5), but not CCI ≥ 1, were independent risk factors for worse OS (Table 6).

Furthermore, we investigated the prognostic factors within the non-R–CHOP group by stratifying patients into the R-chemotherapy group for those receiving chemotherapeutic regimens other than R–CHOP and the non-chemotherapy group for those given single-agent rituximab, steroid monotherapy, or rituximab plus steroid treatment. Notably, a high-risk status (aaIPI ≥ 2) and low GNRI (GNRI ≤ 92.5) were independent risk factors for PFS and OS in both groups. Abnormal serum B2M level only showed significance in the R-chemotherapy group but not in the non-chemotherapy group for PFS and OS. BM involvement at diagnosis was an independent risk factor for PFS in the R-chemotherapy but not in the non-chemotherapy group and it was not an independent factor for OS in both groups (Tables S4 and S5).

## 4. Discussion

In this study, we demonstrated that the nutritional status and comorbidities play important roles in therapeutic modalities and clinical outcomes in newly diagnosed elderly DLBCL patients. Our results revealed that the cutoff value calculated using a ROC curve is predictive of the clinical outcome. This cutoff value is also numerically close to those shown in the previous studies [13,17]. Both CCI and GNRI status stratified all the patients into the favorable and unfavorable prognostic groups, but the CCI status failed to show statistical significance in the multivariate analysis. Notably, our results revealed that the GNRI status was highly predictive regarding survival in the patients who received non-R–CHOP regimens, a substantial proportion of elderly DLBCL patients in a real-world setting [7,8,9].

This study also explored the related factors that may affect initial therapeutic strategies. The results suggested that older age, poorer performance status, lower serum albumin level, and more comorbidities were associated with less intensive treatments (non-R–CHOP regimens). These findings were concordant with previous studies in which palliative chemotherapy, steroid monotherapy, and supportive care were chosen as the initial treatments for unfitness and frailty in real-world scenarios [37,38,39] to avoid anthracycline-related toxicities [40,41].

Our results suggested that low GNRI and aaIPI, rather than the CCI, are independent risk factors for PFS and OS in the non-R–CHOP group after adjustment for various clinical parameters. Among the above markers, the CCI has been a commonly used predictor in elderly DLBCL patients [15]; however, the multivariate analysis in this study failed to show statistical significance. Regarding aaIPI, our study further confirmed it as an influential predictor even in patients stratified by treatment strategies. However, some patients may not complete staging work-ups, resulting in incomplete IPI or aaIPI scores. Our study incorporated the nutritional status into the evaluation, and the results demonstrated that the GNRI, which can be obtained using regular blood tests and body size measurement at diagnosis, is also a feasible prognostic factor, especially in elderly patients receiving non-anthracycline chemotherapy. Collectively, the aaIPI and GNRI scores can be accessed at the time of diagnosis to better predict long-term clinical outcomes in these frail patients.

Some limitations exist in this study. Firstly, because of the retrospective analysis, the results of this study may come with reporting bias. Secondly, only the baseline nutritional status was assessed, without further evaluation of longitudinal assessment of the nutritional status. Thus, the nutritional status during the treatment and its effect on the survival remain to be clarified. Thirdly, we did not classify histologic results into germinal center B cell-like (GCB) type or non-GCB type because of the lack of proper immunohistochemical data from earlier pathologic reports, although a previous study suggested that there was some degree of correlation between the cell of origin and the GNRI regarding survival impacts [17]. Fourth, factors determining whether a patient was in a “fit” or “unfit” condition for receiving anthracycline-containing regimens, including, but not limited to, advanced age, poor performance status, advanced risk status, autoimmune diseases, chronic liver or renal diseases, cardiopulmonary insufficiency, and concurrent second malignancy, should be investigated to determine their roles in the nutritional status and the disease survival.

## 5. Conclusions

In summary, this study investigated the prognostic factors for elderly DLBCL patients. The results revealed that, in addition to the aaIPI, the GNRI score can also serve as an independent prognostic factor of PFS and OS in frail populations. Based on our findings, the nutritional status may play a crucial role regarding the initial treatment evaluation and prognosis. Conclusively, this study provides another feasible prognostic model for unfit elderly DLBCL patients in the real-world setting.

## Figures and Tables

**Table 1:** Baseline characteristics of DLBCL patients according to the treatment group.

	All Patients (*n* = 205)	R–CHOP (*n* = 76)	Non-R–CHOP (*n* = 129)	*p*
Age				
Median, range (years)	75 (65–96)	71 (65–93)	77 (65–96)	<0.001
≥80, *n* (%)	64 (31.2)	10 (13.2)	54 (84.4)	<0.001
70–79, *n* (%)	94 (45.9)	34 (44.7)	60 (46.3)	0.495
65–69, *n* (%)	47 (22.9)	32 (42.1)	15 (11.6)	<0.001
Gender, *n* (%)				
Male	107 (52.2)	46 (60.5)	61 (47.3)	0.091
Female	98 (47.8)	30 (39.5)	68 (57.2)	
ECOG PS, *n* (%)				
≤1	135 (65.9)	66 (86.8)	69 (53.5)	<0.001
≥2	70 (34.1)	10 (13.2)	60 (46.5)	
Ann Arbor stage, *n* (%) *				
I/II	73 (36.3)	34 (44.7)	39 (31.2)	0.074
III/IV	128 (63.7)	42 (55.3)	86 (68.8)	
aaIPI, *n* (%) *				
≤1	93 (47.2)	41 (53.9)	52 (43.0)	0.158
≥2	104 (52.8)	35 (46.1)	69 (57.0)	
Extranodal, *n* (%)	157 (76.6)	54 (71.1)	103 (79.8)	0.151
BM involved, *n* (%)	54 (29.3)	17 (22.7)	37 (33.9)	0.137
B symptoms, *n* (%)	102 (50)	33 (43.4)	69 (53.9)	0.148
LDH				
median, range (IU/dL)	240 (33–3595)	222 (107–1482)	250 (33–3592)	0.139
>ULN, *n* (%)	126 (64)	44 (58.7)	82 (67.2)	0.225
Serum Alb,				
median, range (g/dL)	3.62 (1.76–4.93)	3.79 (1.88–4.93)	3.5 (1.76–4.80)	<0.001
<LLN, *n* (%)	74 (37.8)	15 (20.5)	59 (48.0)	<0.001
Serum Cr,				
median, range (mg/dL)	0.91 (0.11–12.9)	0.90 (0.35–2.9)	0.94 (0.11–5.69)	0.22
CCI ≥ 1	112 (54.9)	28 (36.8)	84 (65.6)	<0.001
CCI ≥ 3	31 (15.2)	5 (6.6)	26 (20.3)	0.008
GNRI score, median, range	94 (40–115)	97 (40–115)	92 (42–113)	<0.001
Low GNRI (≤92.5), *n* (%)	87 (42.6)	20 (26.3)	67 (32.8)	<0.001

* Some patients failed to complete full evaluation of staging (*n* = 4) and aaIPI (*n* = 8); aaIPI, age-adjusted International Prognostic Index; Alb, albumin; BM, bone marrow; Cr, creatinine; CCI, Charlson Comorbidity Index; ECOG PS, Eastern Cooperative Oncology Group Performance Status; GNRI, Geriatric Nutritional Risk Index; LDH, lactate dehydrogenase; LLN, lower limit of normal; R–CHOP, rituximab, cyclophosphamide, doxorubicin, vincristine, and prednisolone; ULN, upper limit of normal.

**Table 2:** Distribution of initial treatments stratified by age and performance status.

Treatment Choices	Age (Years) ^#^			ECOG PS ^#^	
	≥80 (*n* = 64)	70–79 (*n* = 94)	65–69 (*n* = 47)	0–1 (*n* = 135)	2–4 (*n* = 70)
R–CHOP, *n* (%)	10 (15.6)	34 (36.2)	32 (68.1)	66 (48.9)	10 (14.2)
R–COP, *n* (%)	22 (34.4)	41 (43.6)	9 (19.1)	51 (37.8)	21 (30.0)
Other regimens *, *n* (%)	14 (21.9)	4 (4.2)	1 (2.1)	10 (7.4)	9 (12.9)
Steroid alone, *n* (%)	18 (28.1)	15 (16.0)	5 (10.7)	8 (5.9)	30 (42.9)

^#^ Analyzed using the chi-squared test, *p* < 0.001. * Other regimens included the following combinations: rituximab, vincristine, and prednisolone; rituximab and prednisolone. ECOG PS, Eastern Cooperative Oncology Group Performance Status; R–CHOP, rituximab, cyclophosphamide, doxorubicin, vincristine, and prednisolone; R–COP, rituximab, cyclophosphamide, vincristine, and prednisolone.

**Table 3:** Factors associated with receiving non-R–CHOP treatments.

Variables	Univariate Analysis	Multivariate Analysis *		
	OR (95% CI)	*p*	OR (95% CI)	*p*
Age ≥ 80	4.75 (2.24–10.1)	<0.001	3.48 (1.49–8.13)	0.004
Male gender	0.79 (0.53–1.24)	0.168		
PS 2–4	5.74 (2.71–12.2)	<0.001	3.51 (1.44–8.57)	0.006
aaIPI 2–3	1.52 (0.85–2.71)	0.159		
BM involved	1.75 (0.90–3.43)	0.101		
Stage III/IV	1.79 (0.99–3.22)	0.054		
Extranodal	1.61 (0.84–3.11)	0.153		
Abnormal LDH	1.44 (0.80–2.62)	0.226		
Abnormal B2M	3.20 (1.74–5.89)	<0.001	1.02 (0.47–2.21)	0.969
Abnormal Cr	3.53 (1.48–8.43)	0.005	2.32 (0.78–6.89)	0.130
B symptoms	1.52 (0.86–2.70)	0.148		
Low BMI	3.48 (0.75–16.1)	0.111		
Low Alb	3.57 (1.83–6.70)	<0.001	2.59 (1.16–5.79)	0.020
CCI (≥ 1)	3.27 (1.81–5.92)	<0.001	3.08 (1.52–6.23)	0.002

* Factors with a *p*-value less than 0.05 in the univariate analysis were entered into the multivariate analysis; aaIPI, age-adjusted International Prognostic Index; Alb, albumin; B2M, beta-2 microglobulin; BM, bone marrow; BMI, body mass index; CCI, Charlson Comorbidity Index; Cr, creatinine; LDH, lactate dehydrogenase; OR, odds ratio; PS, performance status; R–CHOP, rituximab, cyclophosphamide, doxorubicin, vincristine, and prednisolone.

**Table 4:** Baseline characteristics of DLBCL patients according to the GNRI.

	High GNRI (*n* = 117)	Low GNRI (*n* = 87)	*p*
Age			
Median, range (years)	75 (63–94)	76 (65–95)	0.03
≥80, *n* (%)	30 (25.6)	16 (18.4)	0.058
70–79, *n* (%)	58 (49.6)	36 (41.4)	0.132
65–69, *n* (%)	29 (24.8)	35 (40.2)	0.059
Gender, *n* (%)			
Male	60 (51.3)	47 (54.0)	0.689
Female	57 (48.7)	40 (46.0)	
ECOG PS, *n* (%)			
≤1	92 (78.6)	43 (49.4)	<0.001
≥2	25 (21.4)	44 (50.6)	
Ann Arbor stage, *n* (%) *			
I/II	56 (47.9)	17 (20.2)	<0.001
III/IV	61 (52.1)	67 (79.8)	
aaIPI, *n* (%) *			
≤1	71 (60.7)	22 (27.2)	<0.001
≥2	46 (39.3)	58 (72.5)	
Extranodal, *n* (%)	86 (73.5)	70 (80.5)	0.247
BM involved, *n* (%)	25 (22.5)	29 (39.7)	0.012
B symptoms, *n* (%)	46 (39.7)	55 (63.2)	0.001
LDH			
median, range (IU/dL)	199 (33–2435)	274 (82–3595)	<0.001
>ULN, *n* (%)	61 (53.0)	64 (79.0)	<0.001
Serum Alb,			
median, range (g/dL)	3.89 (3.47–4.93)	3.05 (1.76–3.84)	<0.001
<LLN, *n* (%)	3 (2.6)	70 (89.7)	<0.001
Serum Cr,			
median, range (mg/dL)	0.91 (0.11–8.36)	0.95 (0.53–12.9)	0.207
CCI ≥ 1	64 (54.7)	48 (55.8)	0.875
CCI ≥ 3	18 (15.4)	13 (15.1)	0.958
R–CHOP, *n* (%)	56 (47.9)	20 (23.0)	<0.001

* Some patients failed to complete full evaluation of staging (*n* = 3) and aaIPI (*n* = 7); aaIPI, age-adjusted International Prognostic Index; Alb, albumin; BM, bone marrow; Cr, creatinine; CCI, Charlson Comorbidity Index; ECOG PS, Eastern Cooperative Oncology Group Performance Status; GNRI, Geriatric Nutritional Risk Index; LDH, lactate dehydrogenase; LLN, lower limit of normal; R–CHOP, rituximab, cyclophosphamide, doxorubicin, vincristine, and prednisolone; ULN, upper limit of normal.

**Table 5:** The prognostic factors for PFS according to univariate and multivariate Cox regression.

	R–CHOP Group				Non-R–CHOP Group			
Variables	Univariate Analysis		Multivariate Analysis *		Univariate Analysis		Multivariate Analysis *	
	HR (95% CI)	*p*	HR (95% CI)	*p*	HR (95% CI)	*p*	HR (95% CI)	*p*
Age ≥ 80	1.87 (0.86–4.06)	0.114			1.21 (0.82–1.76)	0.337		
Male gender	1.32 (0.70–2.50)	0.389			1.41 (0.97–2.06)	0.070		
PS 2–4	20.2 (7.78–52.4)	<0.001	14.9 (4.77–46.4)	<0.001	2.76 (1.87–4.07)	<0.001	1.65 (0.92–2.95)	0.092
aaIPI 2–3	2.09 (1.12–3.93)	0.021	1.17 (1.31–1.88)	0.036	3.18 (2.10–4.83)	<0.001	2.70 (1.18–6.27)	0.029
BM involved	1.43 (0.71–2.89)	0.318			2.85 (1.83–4.43)	<0.001	2.50 (1.47–4.25)	0.001
Stage III/IV	1.78 (0.94–3.36)	0.076			2.70 (1.73–4.23)	<0.001	0.99 (0.48–2.04)	0.977
Extranodal	0.84 (0.44–1.60)	0.596			1.03 (0.64–1.63)	0.916		
Abnormal LDH	1.86 (0.96–3.62)	0.066			2.24 (1.46–3.43)	<0.001	0.92 (0.50–1.84)	0.781
Abnormal B2M	1.90 (1.01–3.58)	0.046	1.01 (0.47–2.19)	0.927	2.68 (1.66–4.31)	<0.001	2.98 (1.61–5.53)	0.001
B symptoms	1.43 (0.78–2.64)	0.251			1.73 (1.18–2.53)	0.005	0.96 (0.59–1.57)	0.871
Low Alb	1.39 (0.68–2.86)	0.366			1.75 (1.19–2.58)	0.004	1.81 (0.66–4.97)	0.252
CCI (≥1)	2.30 (1.24–4.27)	0.008	1.58 (0.80–3.11)	0.185	0.88 (0.59–1.32)	0.536		
Low GNRI	1.17 (0.58–2.33)	0.667			1.47 (1.01–2.14)	0.049	2.85 (1.05–7.72)	0.039

* Factors with a *p*-value less than 0.05 in the univariate analysis were entered into the multivariate analysis; aaIPI, age-adjusted International Prognostic Index; Alb, albumin; B2M, beta-2 microglobulin; CCI, Charlson Comorbidity Index; GNRI, Geriatric Nutritional Risk Index; HR, hazard ratio; LDH, lactate dehydrogenase; PS, performance status; R–CHOP, rituximab, cyclophosphamide, doxorubicin, vincristine, and prednisolone.

**Table 6:** The prognostic factors for OS according to univariate and multivariate Cox regression.

	R–CHOP Group				Non-R–CHOP Group			
Variables	Univariate Analysis		Multivariate Analysis *		Univariate Analysis		Multivariate Analysis *	
	HR (95% CI)	*p*	HR (95% CI)	*p*	HR (95% CI)	*p*	HR (95% CI)	*p*
Age ≥ 80	2.08 (0.95–4.56)	0.066			1.30 (0.88–1.92)	0.187		
Male gender	1.38 (0.71–2.65)	0.341			1.48 (1.01–2.18)	0.047	1.36 (0.84–2.19)	0.212
PS 2–4	21.7 (8.31–56.6)	<0.001	15.2 (4.82–47.7)	<0.001	3.22 (2.15–4.82)	<0.001	1.46 (0.78–2.74)	0.243
aaIPI 2–3	2.04 (1.07–3.89)	0.030	1.39 (1.01–1.92)	0.043	3.97 (2.55–6.18)	<0.001	3.08 (1.22–7.77)	0.017
BM involved	1.22 (0.59–2.52)	0.598			3.15 (2.00–4.98)	<0.001	2.69 (1.56–4.65)	<0.001
Stage III/IV	1.80 (0.93–3.46)	0.080			3.18 (1.97–5.13)	<0.001	0.91 (0.42–1.98)	0.819
Extranodal	0.98 (0.50–1.95)	0.962			1.10 (0.68–1.78)	0.710		
Abnormal LDH	1.90 (0.96–3.76)	0.067			2.67 (1.70–4.20)	<0.001	0.99 (0.51–1.90)	0.969
Abnormal B2M	2.23 (1.16–4.29)	0.016	1.25 (0.57–2.74)	0.585	2.67 (1.63–4.37)	<0.001	2.99 (1.54–5.80)	0.001
B symptoms	1.40 (0.75–2.63)	0.291			1.78 (1.20–2.64)	0.004	0.93 (0.55–1.57)	0.790
Low Alb	1.51 (0.73–3.12)	0.263			2.00 (1.34–2.98)	0.001	1.95 (0.66–5.82)	0.229
CCI (≥1)	2.41 (1.28–4.54)	0.007	1.58 (0.78–3.19)	0.202	0.94 (0.62–1.42)	0.750		
Low GNRI	1.28 (0.63–2.57)	0.495			1.62 (1.09–2.41)	0.016	2.98 (1.02–8.90)	0.045

* Factors with a *p*-value less than 0.05 in the univariate analysis were entered into the multivariate analysis; aaIPI, age-adjusted International Prognostic Index; Alb, albumin; B2M, beta-2 microglobulin; CCI, Charlson Comorbidity Index; GNRI, Geriatric Nutritional Risk Index; HR, hazard ratio; LDH, lactate dehydrogenase; PS, performance status; R–CHOP, rituximab, cyclophosphamide, doxorubicin, vincristine, and prednisolone.

## Data Availability

The data presented in this study are available from the corresponding author upon reasonable request.

## Supplementary Materials

The following are available online at https://www.mdpi.com/article/10.3390/nu13093243/s1, Figure S1: ROC analysis according to the GNRI with respect to OS, Table S1: Distribution of initial treatment strategies, Table S2: Classification of causes of death based on therapeutic intervention, Table S3: Investigation of the risk factors of early mortality using univariate and multivariate Cox regression analysis, Table S4: Investigation of the prognostic factors for PFS within non-R-CHOP group by univariate and multivariate Cox regression, Table S5: Investigation of the prognostic factors for OS within non-R-CHOP group by univariate and multivariate Cox regression.

## References

[1] Morton L.M., Wang S.S., Devesa S.S., Hartge P., Weisenburger D.D., Linet M.S. (2006). Lymphoma incidence patterns by WHO subtype in the United States, 1992–2001. Blood.

[2] Dixon D.O., Neilan B., Jones S.E., Lipschitz D.A., Miller T.P., Grozea P.N., Wilson H.E. (1986). Effect of age on therapeutic outcome in advanced diffuse histiocytic lymphoma: The Southwest Oncology Group experience. J. Clin. Oncol..

[3] Vose J.M., Armitage J.O., Weisenburger D.D., Bierman P.J., Sorensen S., Hutchins M., Moravec D.F., Howe D., Dowling M.D., Mailliard J. (1988). The importance of age in survival of patients treated with chemotherapy for aggressive non-Hodgkin’s lymphoma. J. Clin. Oncol..

[4] Coiffier B., Lepage E., Briere J., Herbrecht R., Tilly H., Bouabdallah R., Morel P., Van Den Neste E., Salles G., Gaulard P. (2002). CHOP chemotherapy plus rituximab compared with CHOP alone in elderly patients with diffuse large-B-cell lymphoma. N. Engl. J. Med..

[5] Pfreundschuh M., Trümper L., Osterborg A., Pettengell R., Trneny M., Imrie K., Ma D., Gill D., Walewski J., Zinzani P.L. (2006). CHOP-like chemotherapy plus rituximab versus CHOP-like chemotherapy alone in young patients with good-prognosis diffuse large-B-cell lymphoma: A randomised controlled trial by the MabThera International Trial (MInT) Group. Lancet Oncol..

[6] Coccaro N., Anelli L., Zagaria A., Perrone T., Specchia G., Albano F. (2020). Molecular Complexity of Diffuse Large B-Cell Lymphoma: Can It Be a Roadmap for Precision Medicine?. Cancers.

[7] Cho S.F., Wu W.H., Yang Y.H., Liu Y.C., Hsiao H.H., Chang C.S. (2018). Investigation of treatment pattern, medical resource utilization and demographic prognostic factors in older patients with non-Hodgkin lymphoma: A nationwide population-based study. J. Geriatr. Oncol..

[8] Hershman D.L., McBride R.B., Eisenberger A., Tsai W.Y., Grann V.R., Jacobson J.S. (2008). Doxorubicin, cardiac risk factors, and cardiac toxicity in elderly patients with diffuse B-cell non-Hodgkin’s lymphoma. J. Clin. Oncol..

[9] Tsutsué S., Tobinai K., Yi J., Crawford B. (2020). Nationwide claims database analysis of treatment patterns, costs and survival of Japanese patients with diffuse large B-cell lymphoma. PLoS ONE.

[10] International Non-Hodgkin’s Lymphoma Prognostic Factors Project (1993). A predictive model for aggressive non-Hodgkin’s lymphoma. N. Engl. J. Med..

[11] Carson K.R., Bartlett N.L., McDonald J.R., Luo S., Zeringue A., Liu J., Fu Q., Chang S.H., Colditz G.A. (2012). Increased body mass index is associated with improved survival in United States veterans with diffuse large B-cell lymphoma. J. Clin. Oncol..

[12] Dalia S., Chavez J., Little B., Bello C., Fisher K., Lee J.H., Chervenick P., Sokol L., Sotomayor E., Shah B. (2014). Serum albumin retains independent prognostic significance in diffuse large B-cell lymphoma in the post-rituximab era. Ann. Hematol..

[13] Kanemasa Y., Shimoyama T., Sasaki Y., Hishima T., Omuro Y. (2018). Geriatric nutritional risk index as a prognostic factor in patients with diffuse large B cell lymphoma. Ann. Hematol..

[14] Miura K., Konishi J., Miyake T., Makita M., Hojo A., Masaki Y., Uno M., Ozaki J., Yoshida C., Niiya D. (2017). A Host-Dependent Prognostic Model for Elderly Patients with Diffuse Large B-Cell Lymphoma. Oncologist.

[15] Saygin C., Jia X., Hill B., Dean R., Pohlman B., Smith M.R., Jagadeesh D. (2017). Impact of comorbidities on outcomes of elderly patients with diffuse large B-cell lymphoma. Am. J. Hematol..

[16] Lee S., Fujita K., Morishita T., Negoro E., Oiwa K., Tsukasaki H., Yamamura O., Ueda T., Yamauchi T. (2021). Prognostic utility of a geriatric nutritional risk index in combination with a comorbidity index in elderly patients with diffuse large B cell lymphoma. Br. J. Haematol..

[17] Matsukawa T., Suto K., Kanaya M., Izumiyama K., Minauchi K., Yoshida S., Oda H., Miyagishima T., Mori A., Ota S. (2020). Validation and comparison of prognostic values of GNRI, PNI, and CONUT in newly diagnosed diffuse large B cell lymphoma. Ann. Hematol..

[18] Go S.-I., Kim H.-G., Kang M.H., Park S., Lee G.-W. (2020). Prognostic model based on the geriatric nutritional risk index and sarcopenia in patients with diffuse large B-cell lymphoma. BMC Cancer.

[19] Boyle T., Connors J.M., Gascoyne R.D., Berry B.R., Sehn L.H., Bashash M., Spinelli J.J. (2017). Physical activity, obesity and survival in diffuse large B-cell and follicular lymphoma cases. Br. J. Haematol..

[20] Hong F., Habermann T.M., Gordon L.I., Hochster H., Gascoyne R.D., Morrison V.A., Fisher R.I., Bartlett N.L., Stiff P.J., Cheson B.D. (2014). The role of body mass index in survival outcome for lymphoma patients: US intergroup experience. Ann. Oncol..

[21] Bairey O., Shacham-Abulafia A., Shpilberg O., Gurion R. (2016). Serum albumin level at diagnosis of diffuse large B-cell lymphoma: An important simple prognostic factor. Hematol. Oncol..

[22] Ngo L., Hee S.W., Lim L.C., Tao M., Quek R., Yap S.P., Loong E.L., Sng I., Hwan-Cheong T.L., Ang M.K. (2008). Prognostic factors in patients with diffuse large B cell lymphoma: Before and after the introduction of rituximab. Leuk. Lymphoma.

[23] Camus V., Lanic H., Kraut J., Modzelewski R., Clatot F., Picquenot J.M., Contentin N., Lenain P., Groza L., Lemasle E. (2014). Prognostic impact of fat tissue loss and cachexia assessed by computed tomography scan in elderly patients with diffuse large B-cell lymphoma treated with immunochemotherapy. Eur. J. Haematol..

[24] Lanic H., Kraut-Tauzia J., Modzelewski R., Clatot F., Mareschal S., Picquenot J.M., Stamatoullas A., Leprêtre S., Tilly H., Jardin F. (2014). Sarcopenia is an independent prognostic factor in elderly patients with diffuse large B-cell lymphoma treated with immunochemotherapy. Leuk. Lymphoma.

[25] Marian M., August D.A. (2014). Prevalence of malnutrition and current use of nutrition support in cancer patient study. JPEN J. Parenter. Enter. Nutr..

[26] Eckart A., Struja T., Kutz A., Baumgartner A., Baumgartner T., Zurfluh S., Neeser O., Huber A., Stanga Z., Mueller B. (2020). Relationship of Nutritional Status, Inflammation, and Serum Albumin Levels During Acute Illness: A Prospective Study. Am. J. Med..

[27] Bouillanne O., Morineau G., Dupont C., Coulombel I., Vincent J.P., Nicolis I., Benazeth S., Cynober L., Aussel C. (2005). Geriatric Nutritional Risk Index: A new index for evaluating at-risk elderly medical patients. Am. J. Clin. Nutr..

[28] Lidoriki I., Schizas D., Frountzas M., Machairas N., Prodromidou A., Kapelouzou A., Karavokyros I., Pikoulis E., Kales S.N., Liakakos T. (2021). GNRI as a Prognostic Factor for Outcomes in Cancer Patients: A Systematic Review of the Literature. Nutr. Cancer.

[29] Konishi T., Doki N., Kishida Y., Nagata A., Yamada Y., Kaito S., Kurosawa S., Yoshifuji K., Shirane S., Uchida T. (2019). Geriatric nutritional risk index (GNRI) just before allogeneic hematopoietic stem cell transplantation predicts transplant outcomes in patients older than 50 years with acute myeloid leukemia in complete remission. Ann. Hematol..

[30] Mizuno K., Nakazato T., Ito C., Fujita Y., Ogura S., Kamiya T., Sakurai A., Aisa Y., Mori T. (2019). The prognostic value of geriatric nutritional risk index in patients with follicular lymphoma. Ann. Hematol..

[31] Kaito S., Wada A., Adachi H., Konuma R., Kishida Y., Nagata A., Konishi T., Yamada Y., Kumagai T., Yoshifuji K. (2020). Geriatric nutritional risk index as a useful prognostic factor in second allogeneic hematopoietic stem cell transplantation. Ann. Hematol..

[32] Cho S.F., Liu Y.C., Hsiao H.H., Huang C.T., Tsai Y.F., Wang H.C., Lin S.F., Liu T.C. (2017). Investigation on treatment strategy, prognostic factors, and risk factors for early death in elderly Taiwanese patients with diffuse large B-cell lymphoma. Sci. Rep..

[33] Charlson M.E., Pompei P., Ales K.L., MacKenzie C.R. (1987). A new method of classifying prognostic comorbidity in longitudinal studies: Development and validation. J. Chronic Dis..

[34] Lin T.L., Kuo M.C., Shih L.Y., Dunn P., Wang P.N., Wu J.H., Tang T.C., Chang H., Hung Y.S. (2012). The impact of age, Charlson comorbidity index, and performance status on treatment of elderly patients with diffuse large B cell lymphoma. Ann. Hematol..

[35] Thieblemont C., Grossoeuvre A., Houot R., Broussais-Guillaumont F., Salles G., Traullé C., Espinouse D., Coiffier B. (2008). Non-Hodgkin’s lymphoma in very elderly patients over 80 years. A descriptive analysis of clinical presentation and outcome. Ann. Oncol..

[36] Cheson B.D., Horning S.J., Coiffier B., Shipp M.A., Fisher R.I., Connors J.M., Lister T.A., Vose J., Grillo-López A., Hagenbeek A. (1999). Report of an international workshop to standardize response criteria for non-Hodgkin’s lymphomas. NCI Sponsored International Working Group. J. Clin. Oncol..

[37] Morrison V.A., Hamlin P., Soubeyran P., Stauder R., Wadhwa P., Aapro M., Lichtman S.M. (2015). Approach to therapy of diffuse large B-cell lymphoma in the elderly: The International Society of Geriatric Oncology (SIOG) expert position commentary. Ann. Oncol..

[38] Morrison V.A., Hamlin P., Soubeyran P., Stauder R., Wadhwa P., Aapro M., Lichtman S. (2015). Diffuse large B-cell lymphoma in the elderly: Impact of prognosis, comorbidities, geriatric assessment, and supportive care on clinical practice. An International Society of Geriatric Oncology (SIOG) expert position paper. J. Geriatr. Oncol..

[39] Chaganti S., Illidge T., Barrington S., McKay P., Linton K., Cwynarski K., McMillan A., Davies A., Stern S., Peggs K. (2016). Guidelines for the management of diffuse large B-cell lymphoma. Br. J. Haematol..

[40] Merli F., Luminari S., Rossi G., Mammi C., Marcheselli L., Ferrari A., Spina M., Tucci A., Stelitano C., Capodanno I. (2014). Outcome of frail elderly patients with diffuse large B-cell lymphoma prospectively identified by Comprehensive Geriatric Assessment: Results from a study of the Fondazione Italiana Linfomi. Leuk. Lymphoma.

[41] Wieringa A., Boslooper K., Hoogendoorn M., Joosten P., Beerden T., Storm H., Kibbelaar R.E., Veldhuis G.J., van Kamp H., van Rees B. (2014). Comorbidity is an independent prognostic factor in patients with advanced-stage diffuse large B-cell lymphoma treated with R-CHOP: A population-based cohort study. Br. J. Haematol..

