# Supplementary material for: Low Geriatric Nutritional Risk Index Is Associated with Poorer Prognosis in Elderly Diffuse Large B-Cell Lymphoma Patients Unfit for Intensive Anthracycline-Containing Therapy: A Real-World Study
Source: Nutrients. 2021 Sep 17;13(9):3243. doi: 10.3390/nu13093243 (PMC8468689; doi:10.3390/nu13093243)
Supplement: Supplementary file 1 [file nutrients-13-03243-s001.zip › nutrients-1351586-supplementary.pdf]

Supplemental materials

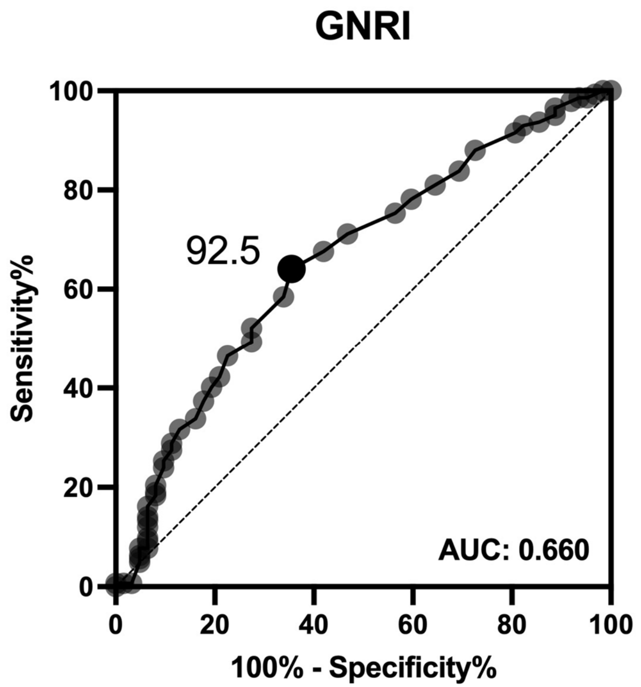

Figure S1. ROC analysis according to GNRI with respect to OS. AUC, area under the curve; GNRI, Geriatric Nutritional Risk Index; ROC, Receiver operating characteristic.

Table S1. Distribution of initial treatment strategies.

Treatment choices	Number of patients, n (%)
R-CHOP	76 (37.1)
R-COP	73 (35.6)
R-OP	3 (1.5)
R-CP	7 (3.4)
BR	2 (1.0)
R or R + steroid	21 (10.2)
Steroid monotherapy	23 (11.2)

R-CHOP, Rituximab, cyclophosphamide, doxorubicin, vincristine, and prednisolone; R-COP, Rituximab, cyclophosphamide, vincristine, and prednisolone; R-OP, Rituximab, vincristine, and prednisolone; R-CP, Rituximab, cyclophosphamide, and prednisolone; BR, Bendamustine and rituximab; R, Rituximab.

Table S2. Classification of causes of death based on therapeutic intervention.

Treatment choices			Main causes of death	
	Lymphoma, n (%)	Infection, n (%)	Toxicity[a], n (%)	Others[b]
R-CHOP, (n = 39)	23 (59.0)	7 (17.9)	7 (17.9)	2 (5.1)
Non-R-CHOP, (n = 104)	61 (58.7)	21 (20.2)	17 (16.3)	5 (4.8)

No significant difference between treatment choices regarding causes of death, $P = 0.989$

[a] Treatment toxicity included neutropenic sepsis, cardiotoxicity, and liver toxicity.

[b] Other causes of death included respiratory failure, cardiac failure, gastrointestinal bleeding, and second malignancy. R-CHOP, rituximab, cyclophosphamide, doxorubicin, vincristine, and prednisolone.

Table S3. Investigation of the risk factors of early mortality using univariate and multivariate Cox regression analysis.

Variables	Univariate analysis		Multivariate analysis*	
	HR 95 % CI	*P* value	HR 95 % CI	*P* value
Age ≥ 80	2.61 (1.58–4.32)	< 0.001	2.58 (1.31–5.11)	0.006
Male gender	1.48 (0.88–2.47)	0.138		
PS 2–4	8.81 (4.90–15.8)	< 0.001	3.00 (0.79–11.2)	0.105
aaIPI 2–3	7.62 (3.44–16.9)	< 0.001	2.57 (1.17–5.67)	0.019
BM involved	3.63 (2.02–6.52)	< 0.001	2.48 (1.25–4.93)	0.010
Stage III / IV	3.67 (1.80–7.48)	< 0.001	0.44 (0.14–1.33)	0.145
Extranodal	1.66 (0.84–3.27)	0.144		
Abnormal LDH	12.1 (3.76–38.6)	< 0.001	2.16 (0.56–8.26)	0.261
Abnormal B2M	12.4 (3.86–39.9)	< 0.001	0.97 (0.56–12.3)	0.783
B symptoms	3.36 (1.90–5.96)	< 0.001	1.56 (0.75–3.23)	0.234
Low BMI	3.65 (2.11–6.31)	< 0.001	0.94 (0.28–3.18)	0.923
CCI (≥ 1)	1.25 (0.75–2.10)	0.398		
Low GNRI (≤ 92)	3.21 (1.88–5.50)	< 0.001	1.06 (0.32–3.51)	0.928

*Factors with a *P* value less than 0.05 in the univariate analysis were entered into the multivariate analysis.

aaIPI, Age-adjusted International Prognostic Index; Alb, Albumin; B2M, Beta2-microglobulin; CCI, Charlson Comorbidity Index; GNRI, Geriatric Nutritional Risk Index; HR, Hazard ratio; LDH, Lactate dehydrogenase; PS, Performance status.

Table S4. Investigation of the prognostic factors for PFS within non-R-CHOP group by univariate and multivariate Cox regression.

Variables	R-chemotherapy group				Non-chemotherapy group			
	Univariate analysis		Multivariate analysis*		Univariate analysis		Multivariate analysis*	
	HR 95 % CI	*P* value	HR 95 % CI	*P* value	HR 95 % CI	*P* value	HR 95 % CI	*P* value
Age ≥ 80	1.09 (0.66–1.79)	0.738			1.05 (0.57–1.93)	0.880		
Male gender	1.32 (0.82–2.14)	0.253			1.52 (0.74–3.38)	0.196		
PS 2–4	2.07 (1.25–3.42)	0.005	1.58 (0.80–3.13)	0.188	1.59 (1.87–4.07)	0.232		
aaIPI 2–3	2.26 (1.38–3.69)	0.001	1.67 (1.04–2.89)	0.041	3.87 (1.13–21.0)	0.034	3.38 (1.28–8.88)	0.014
BM involved	2.08 (1.20–3.63)	0.010	1.81 (0.98–3.35)	0.059	2.18 (0.85–5.60)	0.106		
Stage III / IV	2.32 (1.38–3.90)	0.001	1.43 (0.71–2.86)	0.315	2.02 (0.71–5.77)	0.189		
Extranodal	0.83 (0.47–1.45)	0.508			1.09 (0.46–2.62)	0.841		
Abnormal LDH	1.52 (0.93–2.49)	0.093			8.38 (1.10–63.9)	0.040	0.82 (0.40–16.6)	0.894
Abnormal B2M	2.21 (1.29–3.81)	0.004	2.14 (1.20–3.81)	0.010	6.11 (1.41–26.5)	0.016	4.52 (0.89–23.1)	0.070
B symptoms	1.33 (0.82–2.15)	0.250			2.81 (1.29–6.10)	0.009	2.85 (0.37–22.1)	0.315
Low Alb	1.45 (0.89–2.36)	0.134			1.52 (0.75–3.09)	0.241		
CCI (≥ 1)	0.94 (0.55–1.60)	0.827			1.03 (0.54–1.97)	0.923		
Low GNRI	1.21 (1.24–1.95)	0.045	1.42 (1.04–2.03)	0.049	2.04 (1.71–2.75)	0.036	2.21 (1.66–3.15)	0.025

*Factors with a *P* value less than 0.05 in the univariate analysis were entered into the multivariate analysis. aaIPI, Age-adjusted International Prognostic Index; Alb, Albumin; B2M, Beta2-microglobulin; CCI, Charlson Comorbidity Index; GNRI, Geriatric Nutritional Risk Index; HR, Hazard ratio; LDH, Lactate dehydrogenase; Non-chemotherapy, single-agent rituximab, steroid monotherapy, or rituximab plus steroid treatment; PS, Performance status; R-chemotherapy, chemotherapeutic regimens other than R-CHOP; R-CHOP, rituximab, cyclophosphamide, doxorubicin, vincristine, and prednisolone.

Table S5. Investigation of the prognostic factors for OS within non-R-CHOP group by univariate and multivariate Cox regression.

	R-chemotherapy group				Non-chemotherapy group			
Variables	Univariate analysis		Multivariate analysis*		Univariate analysis		Multivariate analysis*	
	HR 95 % CI	*P* value	HR 95 % CI	*P* value	HR 95 % CI	*P* value	HR 95 % CI	*P* value
Age ≥ 80	1.22 (0.73–2.04)	0.460			1.05 (0.57–1.93)	0.880		
Male gender	1.45 (0.87–2.40)	0.150			1.52 (0.81–2.87)	0.196		
PS 2–4	2.57 (1.52–4.37)	< 0.001	1.65 (0.76–3.58)	0.203	1.59 (0.74–3.38)	0.232		
aaIPI 2–3	2.96 (1.76–4.98)	< 0.001	1.66 (1.12–3.43)	0.036	4.87 (1.13–21.0)	0.034	3.38 (1.28–8.28)	0.014
BM involved	2.33 (1.31–4.17)	0.004	1.95 (1.03–3.70)	0.041	2.18 (0.85–5.60)	0.106		
Stage III / IV	2.86 (1.63–5.02)	< 0.001	1.68 (0.73–3.90)	0.226	2.02 (0.71–5.77)	0.189		
Extranodal	0.89 (0.49–1.62)	0.711			1.09 (0.46–2.62)	0.841		
Abnormal LDH	1.88 (1.12–3.16)	0.017	1.25 (0.59–2.66)	0.558	8.38 (1.10–63.9)	0.040	0.82 (0.40–16.6)	0.894
Abnormal B2M	2.21 (1.25–3.89)	0.006	2.24 (1.07–4.68)	0.032	6.11 (1.41–26.5)	0.016	4.52 (0.89–23.1)	0.070
B symptoms	1.38 (0.84–2.29)	0.206			2.81 (1.29–6.10)	0.009	2.85 (0.37–22.1)	0.315
Low Alb	1.76 (1.06–2.36)	0.030	0.80 (0.43–1.49)	0.484	1.53 (0.75–3.09)	0.241		
CCI (≥ 1)	1.08 (0.61–1.91)	0.788			1.03 (0.54–1.97)	0.923		
Low GNRI	1.42 (1.15–2.06)	0.038	2.43 (1.04–4.01)	0.046	2.09 (1.71–2.75)	0.037	1.77 (1.09–2.12)	0.039

*Factors with a *P* value less than 0.05 in the univariate analysis were entered into the multivariate analysis. aaIPI, Age-adjusted International Prognostic Index; Alb, Albumin; B2M, Beta2-microglobulin; CCI, Charlson Comorbidity Index; GNRI, Geriatric Nutritional Risk Index; HR, Hazard ratio; LDH, Lactate dehydrogenase; Non-chemotherapy, single-agent rituximab, steroid monotherapy, or rituximab plus steroid treatment; PS, Performance status; R-chemotherapy, chemotherapeutic regimens other than R-CHOP; R-CHOP, rituximab, cyclophosphamide, doxorubicin, vincristine, and prednisolone.
